# Hallux Partial Necrosis Associated with Antiphospholipid Syndrome: The Importance of Early Accurate Diagnosis

**DOI:** 10.3390/life13041009

**Published:** 2023-04-13

**Authors:** Antonio Córdoba-Fernández, Francisco Marmol-García, Victoria Eugenia Córdoba-Jiménez

**Affiliations:** 1 Departamento de Podología, Universidad de Sevilla, Avicena Street s/n, 41009 Sevilla, Spain; 2 Área Clínica de Podología, Universidad de Sevilla, Avicena Street s/n, 41009 Sevilla, Spain; franciscojmarmol@hotmail.es; 3 Independent Researcher, Dr. Fleming Street 13, Bajo B, Castilleja de la Cuesta, 41950 Sevilla, Spain; luna.s__16@hotmail.com

**Keywords:** antiphospholipid syndrome, hypercoagulable state, early diagnosis, complication

## Abstract

This study highlights the importance of having a high clinical suspicion of hypercoagulopathy such as antiphospholipid syndrome (APS) in podiatric patients with normal foot pulses and normal standard coagulation tests. APS is an autoimmune disease that is characterized by inflammatory thrombosis in the arteries and veins and obstetric complications such as pregnancy loss. APS usually affects vessels of the lower extremities. We report herein the case of a 46-year-old woman with previous episodes of pre-eclampsia who suffered from partial ischemic necrosis of the hallux of the left foot. After several ischemic episodes of the hallux, with increased risk of toe amputation, the patient was finally diagnosed with APS and treated with specific anticoagulant medication. The patient’s symptoms subsided, and toe amputation was prevented. Early accurate diagnosis and appropriate clinical management are critical to providing optimal outcomes and reducing the risk of amputation.

## 1. Introduction

Antiphospholipid syndrome (APS)is an autoimmune disease that is mediated by autoantibodies and is characterized by thrombosis and pregnancy loss [1]. The hallmarks of the disease are obstetric and vascular complications with inflammatory thrombosis in the arteries and veins [2]. The etiology is unclear, but the intravascular presence of antiphospholipid antibodies (aPL) is suspected to promote the activation of both the endothelium and the complement cascade. As a result, neutrophils and monocytes are recruited into the vascular lumen, and these cells induce activation of coagulation pathways and clot formation [3]. APS can occur alone as a primary disease or in the context of other autoimmune diseases, commonly systemic lupus erythematosus. The rarest presentation of this hypercoagulable condition is the so-called catastrophic APS (CAPS), which is characterized by multiple arterial and/or venous thrombotic events that occur in any organ in a short period of time [4]. Peripheral vascular disease leading to amputation of digits or limbs is a severe complication encountered in patients with APS [5]. We present the case of a 46-year-old woman who consulted for onychocryptosis of the hallux of the left foot with partial necrosis. She referred to previous episodes of ischemic pain that persisted for 6 months as well as pregnancy problems. After laboratory analysis, the patient was finally diagnosed with primary APS, and specific anticoagulant treatment was started, which led to complete resolution of the ischemic process. Thrombotic manifestations of APS are often a therapeutic and diagnostic dilemma and challenge. Despite our increasing knowledge of this disease, many issues remain widely unknown and controversial. This clinical case shows that early accurate diagnosis can be crucial to ensure the best therapy to avoid sequelae.

## 2. Case Report

A 46-year-old woman presented with a three-months evolution of onychocryptosis in the first toe of the left foot. She referred to having suffered several episodes of ischemia in the toe secondary to primary APS that was diagnosed one year ago. She presented with palpable peripheral pulses in both extremities with good capillary refill at distal levels and absence of trophic disorders. The patient reported having suffered from chronic migraines treated with triptyzol and two episodes of pre-eclampsia. The first episode ended with a miscarriage at twenty-two weeks, and the second with the birth of a live preterm infant with brain damage. The patient reported the absence of Raynaud’s phenomenon, autoimmune disease, or thrombotic events.

**Figure 1:**
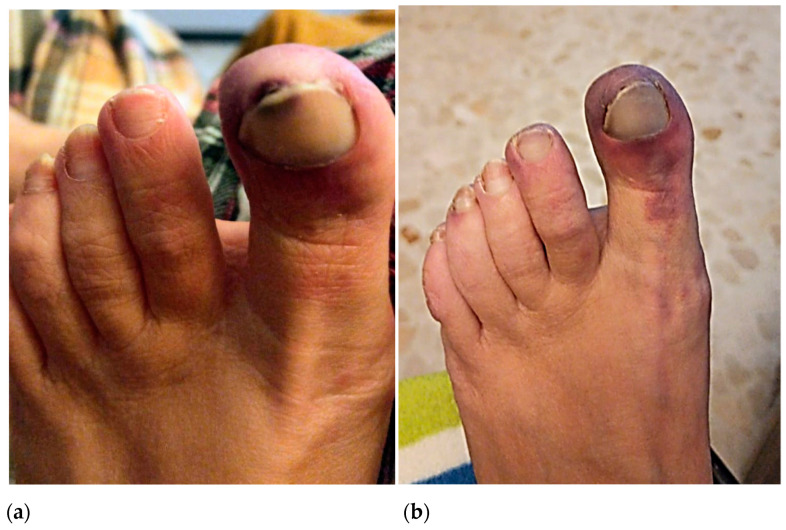
Picture of the first episode where erythema and subungual partial necrosis can be observed in the hallux and the medial edge of the second toe (**a**). Appearance of the hallux in the second episode where the cyanotic appearance of the toe can be observed (**b**).

In October 2020, the patient reported a first episode of erythema and pain on the tip of the big toe and medial edge of the second toe of the left foot, which she initially attributed to poor shoe fit. In January 2021, the patient suffered a second ischemic episode in the big toe of her left foot. Initially, she consulted with a podiatrist who diagnosed a subungual hematoma likely caused by shoe-toe friction (Figure 1a). Due to the gradual worsening of the symptoms and the exacerbated pain associated with the raised extremity, she went to the emergency room of hospitals on several occasions where she was prescribed minor analgesics. In April 2021, after a third episode of intense pain associated with cyanosis in the first toe of the left foot, she went to the hospital where she was evaluated by a dermatologist. Topical application of diltiazem and gentamicin/betamethasone cream (three times daily) was prescribed, and the patient was discharged (Figure 1b).

**Figure 2:**
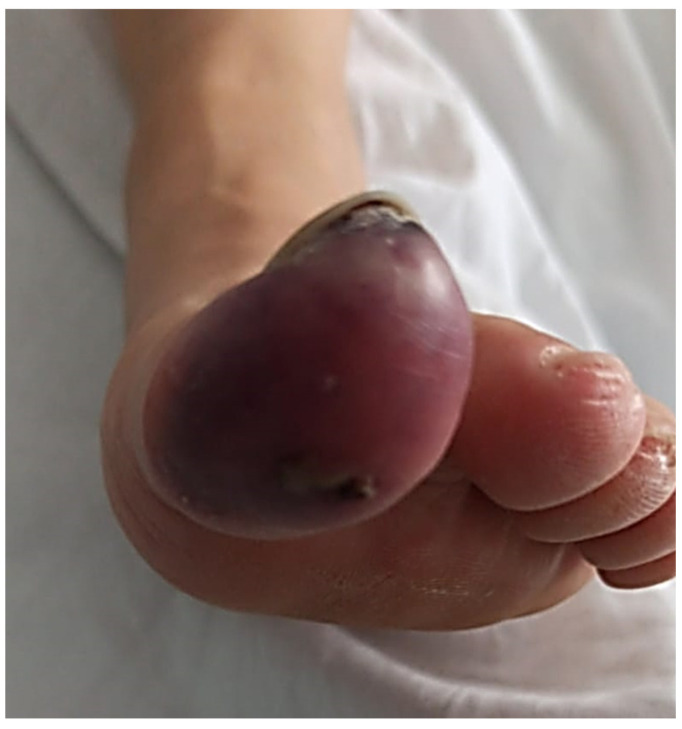
Complete cyanosis with necrosis points in the plantar and the tip level of the first toe can be observed.

Given the progressive worsening of the symptoms, days later she was again examined in the hospital for severe pain, difficulty walking, and signs of erythrocyanosis of the big toe of the left foot (Figure 2). She reported that the pain did not subside with medication and increased when the foot was raised. The emergency room staff reported the presence of cyanosis with decreased capillary refill, dilated erythema, and necrosis at the plantar level and big toe of the left foot. Doppler examination showed the presence of peripheral pulses in both feet without alteration in the arterial flow of the dorsalis pedis and normal ankle-brachial index test. Analgesia was induced with 12 mcg intravenous Toradol, and fentanyl was prescribed daily. She was referred for consultation at the Department of Angiology and Vascular Surgery.

In May 2021, she went to the emergency room again due to an episode of severe pain in the left big toe. Vascular examination showed high blood pressure (184/110 mmHg), heart rate of 87 beats per minute, pedal distal pulses with cyanotic big toe, and absent capillary refill with no signs of infection. No signs of inflammation or distal hypoperfusion of other areas of the affected foot were observed. The laboratory tests revealed a white blood count of 8.290 cells (normal 4000 to 11,000 cells), platelet count 296,000 (normal 130,000 to 450,000), C-reactive protein of 16.6 mg/L (normal 0 to 5.0 mg/L), activated protein C resistance of 4.13 (normal 1.80–10.00), and C1q concentration of 28.7 mg/dL (normal 10.0 to 25.0 mg/dL).The preliminary coagulation study revealed values within a normal platelet count, international normalized ratio or INR (0.90, normal 0.80 to 1.20), activated partial thromboplastin time or APTT (20.4 s, normal range 20–31 s), and functional fibrinogen concentration (4 mg/dL, normal 2 to 5 mg/dL). Diazepam 5 mg was administered for treating hypertension, cilostazol 100 mg, and atorvastatin 40 mg daily were prescribed. Abdominal sonography, CT, and MRI of the lower extremities were requested. Once the patient was hemodynamically stable, she was discharged.

**Figure 3:**
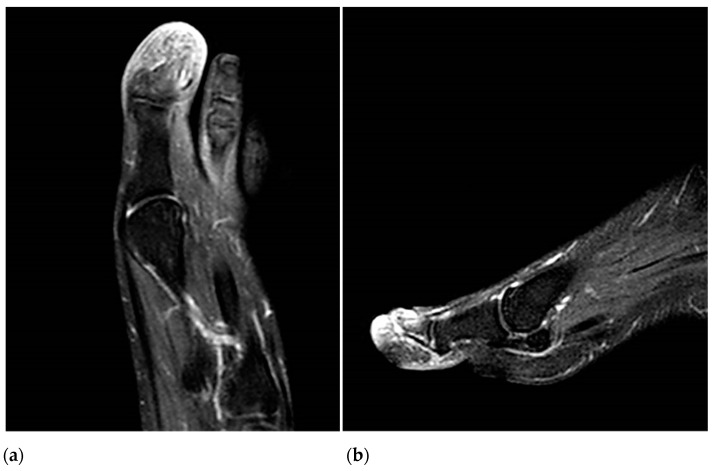
T2-weighted coronal (**a**) and axial (**b**) MRI images show bone edema in the distal phalanx with inflammatory changes in the soft tissues that are more intense in the subungual region.

The CT angiography did not report the presence of stenosis, atheroma, or signs of thrombosis in the arterial system of the affected foot; however, a decrease in the diameter of the posterior tibial artery of the right limb (at the level of the medial malleolus) was observed. There was no filling of the plantar arteries, a fact that does not rule out the presence of thrombosis at this level. Magnetic resonance imaging revealed bone edema with inflammatory changes in the soft tissues of the distal phalanx of the left hallux (Figure 3a,b). Contrast-enhanced magnetic resonance imaging revealed diffuse uptake at the level of a distal phalanx, ruling out the possible existence of a cystic lesion or abscess related to local osteomyelitis. Abdominal ultrasound revealed no organic abnormalities.

**Figure 4:**
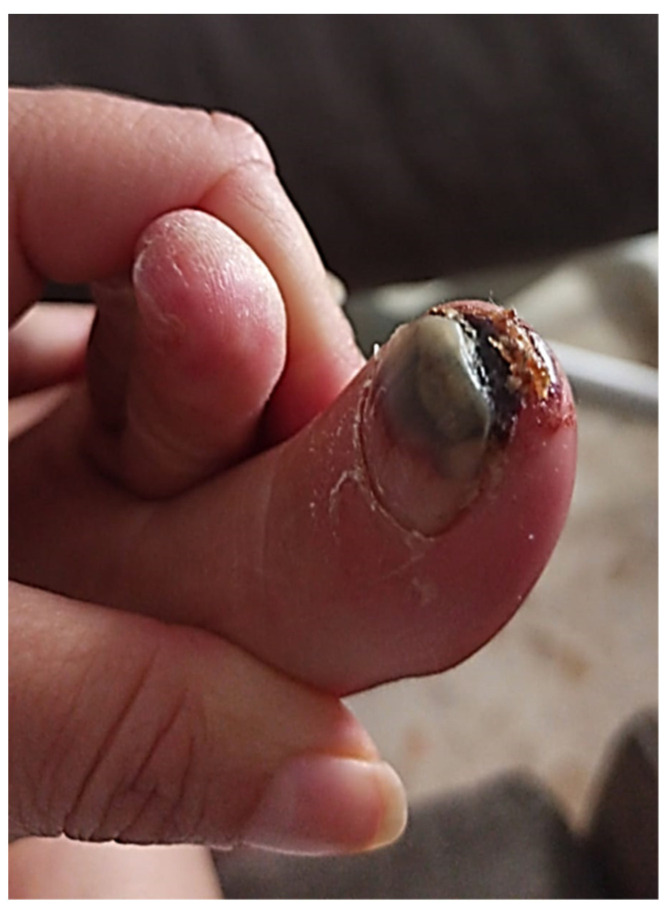
Area of necrosis filed down to the nail bed and tip of the toe and signs of local infection can be observed.

The patient was referred to the Department of Autoimmune Diseases with necrosis in the nail bed and tip of the toe and signs of local infection (Figure 4). Medication with intravenous antibiotics was started. A laboratory dRVVT test revealed the presence of positive lupus anticoagulant (LAC) and positive IgG anti-β2 glycoprotein I (7.5 U/mL; normal < 7). IgM anti-β2 glycoprotein I antibodies (aβ2 GPI) and IgG/ IgM anticardiolipin antibodies (aCL) were normal.

Thrombophilia screening tests were negative or within normal limits except for reduced activity of protein S (58%; normal > 60%). Hence, the diagnosis of APS was suspected. A total of 100 mg of subcutaneous enoxaparin and 25 mg of sildenafil were prescribed daily. After three days of treatment, clinical condition was improved, and the patient was discharged. A small necrosis area remained localized at the distal subungual zone of the first toe. Three months later and prior withdrawal of heparin for 30 h, the hematological tests confirmed the persistence of positive LAC with protein S activity value of 58% (reference 58% to 127%) and protein C value (cromogenic) of 143% (70% to 140%). The diagnosis of primary APS was confirmed. Following medication with anticoagulant drugs, an almost complete resolution of the patient’s symptoms was achieved, and toe amputation was prevented.

At present, the patient fully recovered from the ischemic episode and there were no trophic alterations. Complete healing of the big toe (with only slight hyperesthesia) as well as complete resolution of the chronic migraine episodes were observed. Normal triphasic flow was registered after performing vascular Doppler of the left foot (including the posterior tibial and dorsalis pedis arteries), hallux capillary refill was normal, and blood showed 95% oxygen saturation.

**Figure 5:**
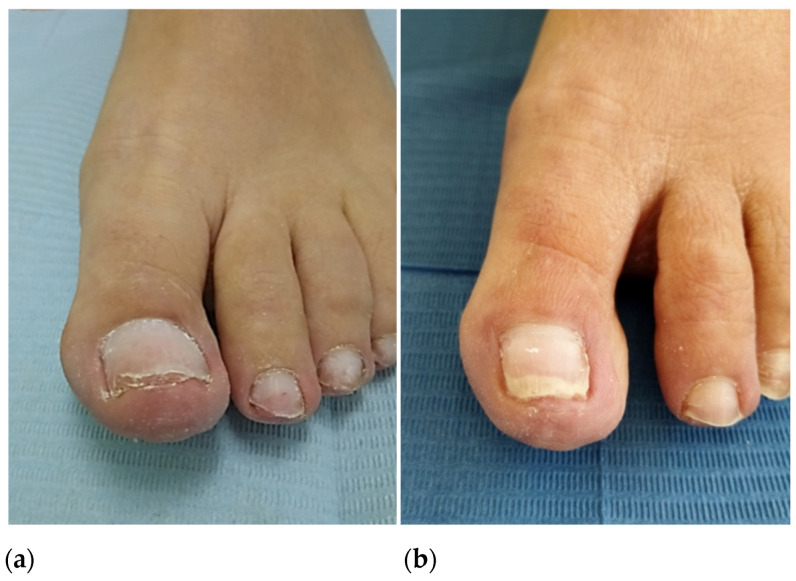
Appearance of the toe a year after the partial ischemic episode (**a**). Postoperative aspect of the toe four months after matricectomy (**b**).

In October 2022, after consultation with the hematologist, the patient underwent segmental phenolization and matricectomy while continuing anticoagulant treatment (enoxaparin 100 mg).The postoperative period was marked by abundant capillary bleeding for four hours, which was controlled with hemostatic gelatin sponges, compression bandage, and elevation of the lower extremity. Over the next months, the healing and recovery time of the nail wounds was as expected and, to date, resolution of the ingrown toenail is complete (Figure 5a,b).

## 3. Discussion

APS is a hypercoagulable disorder in which patients usually present with some type of venous or arterial thrombosis that usually affects the vessels of the lower extremities [5,6,7,8]. APS usually presents alone but can occur in the presence of other autoimmune diseases, commonly systemic lupus erythematosus. APS covers a spectrum of clinical manifestations ranging from recurrent pregnancy loss and obstetric complications to thrombotic disease [9]. In the reported case, the patient had two previous episodes of pre-eclampsia, the first resulting in a 22-week abortion and the second with a live premature newborn, but she was unaware of the presence of APS before the ischemic episode of her foot.

Early accurate clinical diagnosis is crucial to ensure the best therapy and avoid sequelae. In patients with previous episodes of venous or arterial thrombosis in whom APS is suspected, early diagnosis and multidisciplinary management are essential to avoid potentially fatal consequences. An early diagnosis can also prevent the appearance of CAPS, the accelerated presentation form of APS that is characterized by multiple organ failure and very poor prognosis [10,11].

Diagnosis and risk stratification of APS are complex and efforts to standardize and optimize laboratory tests have been ongoing since the initial description of the syndrome. If clinical manifestations are consistent with APS in patients with one or more morbidity events of pregnancy, laboratory tests should be performed to evaluate the presence of antiphospholipid antibodies (aPL). Tests would be aimed at detecting the presence of LAC based on functional coagulation assays, aCL, and aβ2 GPI with immunological assays [12]. Based on the updated classification criteria published in 2006 and called the Sydney criteria, to establish the diagnosis of APS, there must be at least one clinical criterion and one laboratory criterion [12,13].The patient in the present case was initially positive in two of three aPL determinations, but the definitive diagnosis was made with confirmation of LAC positivity in two determinations three months apart.

Antiphospholipid antibodies are thought to activate hemostatic mechanisms through a variety of mechanisms in vivo. The competition of LAC for phospholipid binding that is partially responsible for their in vitro functionality occurs in an artificial system and is not thought to contribute to thrombosis in APS patients. The current consensus is that aPL with LAC activity competes with coagulation factors for binding sites on anionic phospholipids, thus preventing the formation of the enzymatic complexes that drive coagulation [14].Some studies strongly suggest that some aPL binds to C1q and thus initiate an inflammatory response through the classical pathway. This mechanism could be related to the pathophysiological events that lead to thrombosis in patients with APS [15]. In the present case, the elevated levels of C1q initially detected in laboratory tests could be related to the triggering of the pathological process in the patient.

Coagulation blood tests such as PT, INR, APTT, and functional fibrinogen were within normal limits in our patient. Additional laboratory tests revealed the presence of positive LAC and aβ2 GPI. Standard tests allowed us to rule out thrombophilia because of protein C activity, activated protein C resistance, and antithrombin were normal, except for the presence of decreased protein S activity. Prior to withdrawal of heparin, laboratory tests carried out three months after the last ischemic episode confirmed the presence of positive LAC with normal protein S activity.

Some authors have reported that aPL, such as LAC and aCL, inhibit activated protein C and its cofactor, protein S, which explains the relationship between aPL and thrombosis [16]. The formation of the complex between protein S and C4b-binding protein (C4BP) can result in loss of protein S cofactor function that acts as a bridge between coagulation and inflammation due to the involvement of C4BP in regulating complement activation [17]. Whilst it is correct that in our patient, the slightly reduced protein S activity could be due to the acute phase response, acquired reduction in protein S activity could also occur due to the presence of aPL. Given the protein S normalized but the LAC persisted, it is therefore more likely the reduced protein S activity was acute-phase related. We believe that this circumstance points to a transient situation that may have contributed to the patient’s condition.

Headache episodes have been frequently reported in association with APS. Several studies reported high-aPL levels in primary and secondary APS migraineurs. In patients with APS and migraines, serologic and coagulation studies are strongly recommended. Many cases of severe refractory migraines, as reported in this paper, resolved well with anticoagulant therapy [18].

In the absence of histopathology, it may be difficult to distinguish APL-related thrombosis of another concomitant atherosclerotic occlusions from vasculitis of peripheral vessels, and clinicians must be guided purely by clinical presentation [19].The available evidence shows that subclinical changes in the lower extremities arteries in patients with APS are more common than in healthy subjects, and 64-slice CT-angiography with contrast is the method of choice for monitoring disease progression [20]. In the present case, the CT angiography did not show stenosis, atheroma plaques, or signs of thrombosis in the arterial system of the affected foot; however, a drastic decrease in the caliber of the posterior tibial artery of the right extremity was observed without filling in the plantar arteries, although without clinical manifestation.

The medical treatment of APS typically includes aggressive anticoagulation to prevent recurrent thrombosis. In the absence of histopathologic findings, APS can be difficult to distinguish from other atherosclerotic occlusions or vasculitis related to peripheral vessel thrombosis. In such cases, low-molecular-weight heparin and unfractionated heparin can be used initially; however, for long-term treatment, warfarin and vitamin K antagonists are the medication of first choice for most APS patients. It should be noted that warfarin is contraindicated for pregnant women. A recent meta-analysis suggests that vitamin K antagonists remain the first treatment option for high-risk APS patients, being a more appropriate anticoagulant option than direct oral anticoagulants [21]. Commercially available direct anticoagulants may exhibit different levels of efficacy and safety for thromboprophylaxis in APS, a fact which requires further exploration [22]. Although the patient initially refused to receive oral treatment with vitamin K antagonists, once informed of the long-term risk of low-molecular-weight heparin treatment, she agreed to be treated with acenocoumarol.

Appropriate perioperative management is imperative in these patients because life- and limb-threatening complications can occur postoperatively despite aggressive anticoagulation. The surgical management of the patients with APS should be performed as if they had vascular thrombosis, and perioperative anticoagulation should be maintained [7]. As in the present case, in minor surgical procedures, the application of topical hemostatic devices such as gelatin sponges has been shown to be effective. Gelatin sponges can be very useful alone or in combination with antifibrinolytic agents in patients undergoing nail surgery and who are treated with pharmacological drugs that inhibit blood coagulation [23].

In conclusion, APS can be a devastating disease with a wide range of sequelae, many of which often manifest in the lower extremities. In the present case, we present a patient with a history of partial ischemic gangrene of the big toe that is secondary to APS with normal vascular pulses. This case highlights the importance of having a high clinical suspicion of hypercoagulopathy such as APS with normal foot pulses and normal standard coagulation tests. Early accurate diagnosis may be crucial to control the disease and avoid sequelae. In cases where surgery is indicated, the disease must be adequately controlled and the patient must undergo surgery without discontinuing the anticoagulant medication, especially if the lower extremities are affected.

## Data Availability

Not applicable.
